# Supplementary material for: Resolving the full spectrum of human genome variation using Linked-Reads
Source: Genome Res. 2019 Apr;29(4):635–45. doi: 10.1101/gr.234443.118 (PMC6442396; doi:10.1101/gr.234443.118)
Supplement: Supplemental Material [file supp_gr.234443.118_Supplemental_longranger-2.2.zip › longranger-2.2.tar.bz2!/lr2.2-public/lib/go/src/code.google.com/vitess/docs/user-guide/scalability-philosophy.html]

Vitess / Scalability Philosophy


Toggle navigation

- Overview
- Guides
- Reference
- Blog
- GitHub

- #### Overview

  - What is Vitess
  - Scaling MySQL with Vitess
  - Key Concepts
- #### Getting Started

  - Run Vitess on Kubernetes
    - Custom Docker Build
  - Run Vitess Locally
- #### User Guide

  - Introduction- Client Libraries- Backing Up Data- Reparenting
        - Schema Management
        - VSchema Guide
        - Schema Swap (Tutorial)
        - Sharding
          - Horizontal Sharding (Tutorial, automated)
          - Horizontal Sharding (Tutorial, manual)
          - Sharding in Kubernetes (Tutorial, automated)
          - Sharding in Kubernetes (Tutorial, manual)
        - Vitess Sequences
        - MySQL Server Protocol
        - Vitess and Replication
        - Update Stream
        - Row Based Replication
        - Topology Service
        - Transport Security Model
        - Launching
          - Scalability Philosophy
          - Production Planning
          - Server Configuration
          - 2PC Guide
          - Troubleshooting
        - Upgrading
- #### Reference Guides

  - Vitess API- vtctl Commands
- #### Other Resources

  - Presentations- Blog- Roadmap
- #### Contributing

  - Contributing to Vitess- GitHub Workflow- Code Reviews
- GitHub

# Scalability Philosophy

- #### Overview

  - What is Vitess
  - Scaling MySQL with Vitess
  - Key Concepts
- #### Getting Started

  - Run Vitess on Kubernetes
    - Custom Docker Build
  - Run Vitess Locally
- #### User Guide

  - Introduction- Client Libraries- Backing Up Data- Reparenting
        - Schema Management
        - VSchema Guide
        - Schema Swap (Tutorial)
        - Sharding
          - Horizontal Sharding (Tutorial, automated)
          - Horizontal Sharding (Tutorial, manual)
          - Sharding in Kubernetes (Tutorial, automated)
          - Sharding in Kubernetes (Tutorial, manual)
        - Vitess Sequences
        - MySQL Server Protocol
        - Vitess and Replication
        - Update Stream
        - Row Based Replication
        - Topology Service
        - Transport Security Model
        - Launching
          - Scalability Philosophy
          - Production Planning
          - Server Configuration
          - 2PC Guide
          - Troubleshooting
        - Upgrading
- #### Reference Guides

  - Vitess API- vtctl Commands
- #### Other Resources

  - Presentations- Blog- Roadmap
- #### Contributing

  - Contributing to Vitess- GitHub Workflow- Code Reviews

Scalability problems can be solved using many approaches. This document describes Vitess’ approach to address these problems.

## Small instances

When deciding to shard or break databases up into smaller parts, it’s tempting to break them just enough that they fit in one machine. In the industry, it’s common to run only one MySQL instance per host.

Vitess recommends that instances be broken up to be even smaller, and not to shy away from running multiple instances per host. The net resource usage would be about the same. But the manageability greatly improves when MySQL instances are small. There is the complication of keeping track of ports, and separating the paths for the MySQL instances. However, everything else becomes simpler once this hurdle is crossed.

There are fewer lock contentions to worry about, replication is a lot happier, production impact of outages become smaller, backups and restores run faster, and a lot more secondary advantages can be realized. For example, you can shuffle instances around to get better machine or rack diversity leading to even smaller production impact on outages, and improved resource usage.

### Cluster orchestration

Vitess started on baremetal at YouTube, and some still choose to run it that way.
But running Vitess in a cluster orchestration system is the key to achieving the
benefits of small instances without adding management overhead for each new instance.

We provide sample configs to help you get started on Kubernetes
since it's the most similar to Borg (the predecessor to Kubernetes
on which Vitess now runs in YouTube).
If you're more familiar with alternatives like Mesos, Swarm, Nomad, or DC/OS,
we'd welcome your contribution of sample configs for Vitess.

These orchestration systems typically use containers
to isolate small instances so they can be efficiently packed onto machines
without contention on ports, paths, or compute resources.
Then an automated scheduler does the job of shuffling instances around for
failure resilience and optimum utilization.

## Durability through replication

Traditional data storage software treated data as durable as soon as it was flushed to disk. However, this approach is impractical in today’s world of commodity hardware. Such an approach also does not address disaster scenarios.

The new approach to durability is achieved by copying the data to multiple machines, and even geographical locations. This form of durability addresses the modern concerns of device failures and disasters.

Many of the workflows in Vitess have been built with this approach in mind. For example, turning on semi-sync replication is highly recommended. This allows Vitess to failover to a new replica when a master goes down, with no data loss. Vitess also recommends that you avoid recovering a crashed database. Instead, create a fresh one from a recent backup and let it catch up.

Relying on replication also allows you to loosen some of the disk-based durability settings. For example, you can turn off sync\_binlog, which greatly reduces the number of IOPS to the disk thereby increasing effective throughput.

## Consistency model

Distributing your data has its tradeoffs. Before sharding or moving tables to different keyspaces, the application needs to be verified (or changed) such that it can tolerate the following changes:

- Cross-shard reads may not be consistent with each other.
- Cross-shard transactions can fail in the middle and result in partial commits. There is a proposal out to make distributed transactions complete atomically, and on Vitess’ roadmap; however, that is not implemented yet.

Single shard transactions continue to remain ACID, just like MySQL supports it.

If there are read-only code paths that can tolerate slightly stale data, the queries should be sent to REPLICA tablets for OLTP, and RDONLY tablets for OLAP workloads. This allows you to scale your read traffic more easily, and gives you the ability to distribute them geographically.

This tradeoff allows for better throughput at the expense of stale or possible inconsistent reads, since the reads may be lagging behind the master, as data changes (and possibly with varying lag on different shards). To mitigate this, VTGates are capable of monitoring replica lag and can be configured to avoid serving data from instances that are lagging beyond X seconds.

For true snapshot, the queries must be sent to the master within a transaction. For read-after-write consistency, reading from the master without a transaction is sufficient.

To summarize, these are the various levels of consistency supported:

- REPLICA/RDONLY read: Servers be scaled geographically. Local reads are fast, but can be stale depending on replica lag.
- MASTER read: There is only one worldwide master per shard. Reads coming from remote locations will be subject to network latency and reliability, but the data will be up-to-date (read-after-write consistency). The isolation level is READ\_COMMITTED.
- MASTER transactions: These exhibit the same properties as MASTER reads. However, you get REPEATABLE\_READ consistency and ACID writes for a single shard. Support is underway for cross-shard Atomic transactions.

### No multi-master

Vitess doesn’t support multi-master setup. It has alternate ways of addressing most of the use cases that are typically solved by multi-master:

- Scalability: There are situations where multi-master gives you a little bit of additional runway. However, since the statements have to eventually be applied to all masters, it’s not a sustainable strategy. Vitess addresses this problem through sharding, which can scale indefinitely.
- High availability: Vitess integrates with Orchestrator, which is capable of performing a failover to a new master within seconds of failure detection. This is usually sufficient for most applications.
- Low-latency geographically distributed writes: This is one case that is not addressed by Vitess. The current recommendation is to absorb the latency cost of long-distance round-trips for writes. If the data distribution allows, you still have the option of sharding based on geographic affinity. You can then setup masters for different shards to be in different geographic location. This way, most of the master writes can still be local.

### Big data queries

There are two main ways to access the data for offline data processing (as
opposed to online web or direct access to the live data): sending queries to
rdonly servers, or using a Map Reduce framework.

#### Batch queries

These are regular queries, but they can consume a lot of data. Typically, the
streaming APIs are used, to consume large quantities of data.

These queries are just sent to the *rdonly* servers (also known as *batch*
servers). They can take as much resources as they want without affecting live
traffic.

#### MapReduce

Vitess supports MapReduce access to the data. Vitess provides a Hadoop
connector, that can also be used with Apache Spark. See the Hadoop package
documentation
for more information.

With a MapReduce framework, Vitess does not support very complicated
queries. In part because it would be difficult and not very efficient, but also
because the MapReduce frameworks are usually very good at data processing. So
instead of doing very complex SQL queries and have processed results, it is
recommended to just dump the input data out of Vitess (with simple *select*
statements), and process it with a MapReduce pipeline.

## Multi-cell

Vitess is meant to run in multiple data centers / regions / cells. In this part,
we'll use *cell* as a set of servers that are very close together, and share the
same regional availability.

A cell typically contains a set of tablets, a vtgate pool, and app servers that
use the Vitess cluster. With Vitess, all components can be configured and
brought up as needed:

- The master for a shard can be in any cell. If cross-cell master access is
  required, vtgate can be configured to do so easily (by passing the cell that
  contains the master as a cell to watch).
- It is not uncommon to have the cells that can contain the master be more
  provisioned than read-only serving cells. These *master-capable* cells may
  need one more replica to handle a possible failover, while still maintaining
  the same replica serving capacity.
- Failing over from one master in one cell to a master in a different cell is no
  different than a local failover. It has an implication on traffic and latency,
  but if the application traffic also gets re-directed to the new cell, the end
  result is stable.
- It is also possible to have some shards with a master in one cell, and some
  other shards with their master in another cell. vtgate will just route the
  traffic to the right place, incurring extra latency cost only on the remote
  access. For instance, creating U.S. user records in a database with masters in
  the U.S. and European user records in a database with masters in Europe is
  easy to do. Replicas can exist in every cell anyway, and serve the replica
  traffic quickly.
- Replica serving cells are a good compromise to reduce user-visible latency:
  they only contain *replica* servers, and master access is always done
  remotely. If the application profile is mostly reads, this works really well.
- Not all cells need *rdonly* (or batch) instances. Only the cells that run
  batch jobs, or MapReduce jobs, really need them.

Note Vitess uses local-cell data first, and is very resilient to any cell going
down (most of our processes handle that case gracefully).

## Lock server

Vitess is a highly available service, and Vitess itself needs to store a small
amount of metadata very reliably. For that purpose, Vitess needs a highly
available and consistent data store.

Lock servers were built for this exact purpose, and Vitess needs one such
cluster to be setup to run smoothly. Vitess can be customized to utilize any
lock server, and by default it supports zookeeper and etcd. We call this
component
Topology Service.

As Vitess is meant to run in multiple data centers / regions (called cells
below), it relies on two different lock servers:

- global instance: it contains global meta data, like the list of Keyspaces /
  Shards, the VSchema, ... It should be reliable and distributed across multiple
  cells. Running Vitess processes almost never access the global instance.
- per-cell instance (local): It should be running only in the local cell. It
  contains aggregates of all the global data, plus local running tablet
  information. Running Vitess processes get most of their topology data from the
  local instance.

This separation is key to higher reliability. A single cell going bad is never
critical for Vitess, as the global instance is configured to survive it, and
other cells can take over the production traffic. The global instance can be
unavailable for minutes and not affect serving at all (it would affect VSchema
changes for instance, but these are not critical, they can wait for the global
instance to be back).

If Vitess is only running in one cell, both global and local instances can share
the same lock service instance. It is always possible to split them later when
expanding to multiple cells.

## Monitoring

The most stressful part of running a production system is the situation where one is trying to troubleshoot an ongoing outage. You have to be able to get to the root cause quickly and find the correct remedy. This is one area where monitoring becomes critical and Vitess has been battle-tested. A large number of internal state variables and counters are continuously exported by Vitess through the /debug/vars and other URLs. There’s also work underway to integrate with third party monitoring tools like Prometheus.

Vitess errs on the side of over-reporting, but you can be picky about which of these variables you want to monitor. It’s important and recommended to plot graphs of this data because it’s easy to spot the timing and magnitude of a change. It’s also essential to set up various threshold-based alerts that can be used to proactively prevent outages.

## Development workflow

Vitess provides binaries and scripts to make unit testing of the application
code very easy. With these tools, we recommend to unit test all the application
features if possible.

A production environment for a Vitess cluster involves a topology service,
multiple database instances, a vtgate pool and at least one vtctld process,
possibly in multiple data centers. The vttest library uses the *vtcombo* binary
to combine all the Vitess processes into just one. The various databases are
also combined into a single MySQL instance (using different database names for
each shard). The database schema is initialized at startup. The (optional)
VSchema is also initialized at startup.

A few things to consider:

- Use the same database schema in tests as the production schema.
- Use the same VSchema in tests as the production VSchema.
- When a production keyspace is sharded, use a sharded test keyspace as
  well. Just two shards is usually enough, to minimize test startup time, while
  still re-producing the production environment.
- *vtcombo* can also start the *vtctld* component, so the test environment is
  visible with the Vitess UI.
- See
  vttest.proto
  for more information.

## Application query patterns

Although Vitess strives to minimize the app changes required to scale,
there are some important considerations for application queries.

### Bind variables

We strongly recommend using bind variables for all data values in a query.
In addition to being more secure (you don't need to worry about escaping
bind variable values), this allows Vitess to recognize queries that come from
the same code path in your app. Vitess can then cache the execution plan for
that query, instead of recomputing it every time you send different values.

This is similar to prepared statements in MySQL, and in fact that's how you
would use bind variables with Vitess through a connector like JDBC or PDO.
The difference is that Vitess connectors do not communicate with the server
to prepare a statement. They just create a client-side object that wraps the
query and bind variables so they can be sent together over the Vitess RPC
interface.

Note that bind variables are required when sending binary data, since the
Vitess RPC interface requires the query itself to be valid UTF-8.

### Tablet types

Since Vitess handles query routing for you and lets you access any
instance in the cluster from any single VTGate endpoint,
the Vitess clients have an additional parameter for you to specify
which tablet type you want
to send your query to.

Writes must be directed to a *master* type tablet, as well as reads
that should remain part of a larger write transaction.
You also may want to read from the master if there are queries that
must return the most up-to-date value possible, such as when reading
a row that was just modified.

Reads that can tolerate a small amount of replication lag should
target *replica* type tablets. This allows you to scale your read
traffic separately from writes by adding more replicas without
needing to add more shards. Tablets of the *replica* type are
candidates for being promoted to master, so it's important to
define an operational policy that prevents them from becoming so
overloaded that they fall behind on replication by more than a
few seconds (which would make failovers slow).

The *rdonly* tablet type defines a separate pool of slaves
that are ineligible to become master. The separation makes it
safe to allow these instances to get behind on replication
(such as while executing expensive analytic queries)
or have replication stopped altogether (when taking backups
or clones for resharding).

### Query support

A sharded Vitess is not 100% backward compatible with MySQL.
Some queries that used to work will cease to work.
It’s important that you run all your queries on a sharded test environment -- see the Development workflow section above -- to make sure none will fail on production.

Our goal is to expand query support based on the needs of users.
If you encounter an important construct that isn't supported,
please create or comment on an existing feature request so we
know how to prioritize.

Contact: vitess@googlegroups.com  **·**  
Announcements  **·**  
© 2017 Vitess powered by Google Inc
